# Self injuries in adolescence, an unusual clinical presentation of autism

**DOI:** 10.1192/j.eurpsy.2022.1111

**Published:** 2022-09-01

**Authors:** A. Bermejo Pastor, M. Gascón González, M. Jiménez Cabañas, B. Rodado León, A. García Carpintero, R. Pérez Moreno

**Affiliations:** 1 Hospital Clínico San Carlos, Instituto De Psiquiatría Y Salud Mental, Madrid, Spain; 2 Hospital Clínico de Santiago, Servicio De Psiquiatría, Santiago de Compostela, Spain

**Keywords:** Adolescents, Autism Spectrum Disorder, Female autism phenotipe

## Abstract

**Introduction:**

Although autism is only twice more common in men than women in general population, in clinical samples women are underrepresented. This difference may be due to a poor sensitivity of current diagnostic criteria of autism related to females. We present a 13-year-old woman referred to the adolescent psychiatric unit for anxiety, self injuries and suicidal ideation. After careful assessment of current symptoms and neurodevelopmental milestones, deficits in emotional-comunicational reciprocity, nonverbal comunication and relationships emerged, as well as inflexible adherence to routines and restricted interests. The diagnose of autism spectrum disorder was made and the patient started a specific treatment.

**Objectives:**

To review the clinical features of autism spectrum disorders in adolescent females and its differential diagnosis.

**Methods:**

Review of the literature on autism spectrum disorders in female and its specific features.

**Results:**

The “Female Autism Phenotype” is a group features that are more common in autistic women, as opposed to the classic symptoms of autism in men. Some of these differential characteristics are: fewer social impairments and higher levels of social motivation; more age and gender appropriate restricted and repetitive interests; more internalizing rather than externalizing symptoms; and a tendency towards camouflaging

**Conclusions:**

- Autism in women is frequently underdiagnosed. - Females express autism in ways that not allways meet the current diagnostic criteria. - The “Female Autism Phenotype” has been proposed as an specific way of expression of autism in females.

**Disclosure:**

No significant relationships.

